# Extirpation of a Voluminous Bronchocele

**Published:** 1851-11

**Authors:** 


*Extirpation of a Voluminous Bronchocele.* By M. Roux.—M. Roux presented, some time ago, a patient of his to the Academy of Medicine, from whom he had successfully removed a large bronchocele. The man's age is about thirty-five, and the tumor, at first very small, had been increasing, during the last fifteen years, to the size of two fists, and lay principally on the left side of the neck. The swelling was somewhat hard, almost immoveable; and seemed firmly attached to the larynx. The patient was extremely anxious for its removal, and M. Roux, after some hesitation, consented to operate. The incision was made in a longitudinal direction, and ran from the os hyoides to the sternum; M. Roux found it easy to enucleate the growth, with the precaution, however, of tying the thyroid arteries and some venous trunks as they came into view. When the tumour was almost completely detached, the pedicle, by which it was still connected with the neck, was tied, and then divided in front of the ligature.

The only phenomenon worthy of attention, which was noticed during the operation, was a fit of dyspnœa, after which the patient experienced an almost complete aphony; this lasted some time after the operation, and even now there is much hoarseness left. M. Roux accounts for this circumstance by attributing it to the division of the anterior laryngeal nerve. The patient was operated on two months ago, and the wound is now perfectly cicatrized. The tumor weighed about nine ounces and a half; it measured nine inches in circumference, and about seven in its transverse diameter. M. Velpeau was afraid that M. Roux's example would be too readily followed by other surgeons under less favorable circumstances, and thought the operation very dangerous. At the meeting of the 24th of September, M. Velpeau himself brought forward another case of extirpation of the bronchocele, which was performed by M. Cabaret, of St. Malo; this was successful, as well as a third undertaken by M. Hutin. Some discussion arose as to the propriety of giving the name of goître to tumors arising *on* the thyroid body, as well as to enlargement of that gland itself. M. Sédillot contended that the appellation ought to be confined to enlargements of the thyroid gland; Messrs. Velpeau and Roux opposed that opinion.—*London Lancet.*

